# Evaluating the Impact of Novel Incretin Therapies on Cardiovascular Outcomes in Type 2 Diabetes: An Early Systematic Review

**DOI:** 10.3390/ph17101322

**Published:** 2024-10-03

**Authors:** Teodor Salmen, Claudia-Gabriela Potcovaru, Ioana-Cristina Bica, Rosaria Vincenza Giglio, Angelo Maria Patti, Roxana-Adriana Stoica, Marcello Ciaccio, Mohamed El-Tanani, Andrej Janež, Manfredi Rizzo, Florentina Gherghiceanu, Anca Pantea Stoian

**Affiliations:** 1 Doctoral School, “Carol Davila” University of Medicine and Pharmacy, 050474 Bucharest, Romaniaclaudia-gabriela.potcovaru@drd.umfcd.ro (C.-G.P.); ioana-cristina.bica@drd.umfcd.ro (I.-C.B.); roxana-adriana.stoica@drd.umfcd.ro (R.-A.S.); 2 Department of Biomedicine, Neuroscience and Advanced Diagnostics, University of Palermo, 90133 Palermo, Italy; rosariavincenza.giglio@unipa.it (R.V.G.); marcello.ciaccio@unipa.it (M.C.); 3 Department of Laboratory Medicine, University Hospital, 90133 Palermo, Italy; 4 Internal Medicine Unit, “Vittorio Emanuele II” Hospital, 91022 Castelvetrano, Italy; pattiangelomaria@gmail.com; 5 College of Pharmacy, Ras Al Khaimah Medical and Health Sciences University, Ras Al Khaimah 11172, United Arab Emirates; eltanani@rakmhsu.ac.ae (M.E.-T.); manfredi.rizzo@unipa.it (M.R.); 6 Department of Endocrinology, Diabetes and Metabolic Diseases, University Medical Center Ljubljana, 1000 Ljubljana, Slovenia; andrej.janez@kclj.si; 7 School of Medicine, Promise Department of Health Promotion Sciences Maternal and Infantile Care, Internal Medicine and Medical Specialties, University of Palermo, 90133 Palermo, Italy; 8 Department of Marketing and Medical Technology, “Carol Davila” University of Medicine and Pharmacy, 050474 Bucharest, Romania; 9 Department of Diabetes, Nutrition and Metabolic Disease, “Carol Davila” University of Medicine and Pharmacy, 050474 Bucharest, Romania; anca.stoian@umfcd.ro

**Keywords:** retatrutide, tirzepatide, type 2 diabetes mellitus, cardiovascular disease

## Abstract

*Background* This systematic review is registered with CRD42024507397 protocol number and aims to compare the known data about retatrutide on long-term cardiovascular (CV) protection with tirzepatide, an incretin with recent proven CV benefits. *Material and Methods* The inclusion criteria were (i) original full-text articles that are randomized control or clinical trials; (ii) published within the last ten years; (iii) published in English; and (iv) conducted on adult human populations. The exclusion criteria were articles deruled on cell cultures or mammals. Studies were selected if they (1) included patients with type 2 diabetes mellitus (DM) and CV risk; (2) patients that received either tirzepatide or retatrutide; and (3) provided sufficient information such as the corresponding 95% confidence intervals or at least a sufficient *p*-value. Studies were excluded if they were a letter to the editor, expert opinions, case reports, meeting abstracts, or reviews; redundant publications; or needed more precise or complete data. *Results* The seven included studies were assessed for bias with the Newcastle Ottawa scale, heterogenous, and emphasized the potential CV beneficial effect of type 2 DM (T2DM) therapies (glycemia, glycated A1c hemoglobin, body weight, lipid profile, blood pressure and renal parameter). *Discussions* Further, longer follow-up studies are necessary to verify the long-term CV protection, standardize the specific aspects of CV risk, and compare with subjects without T2DM for a more integrative interpretation of the CV effects independent of the improvement of metabolic activity.

## 1. Introduction

Diabetes mellitus (DM), whose hallmark is elevated blood sugar, is a health challenge, with a growing number of new cases of type 2 DM (T2DM) worldwide contributing to the pandemic of non-communicable chronic diseases. As a major threat to vascular health, T2DM increases the risk of cardiovascular disease (CVD) and also affects various organs, including the eyes, kidneys, and nerves. Damage to these organs results in deficits associated with diabetes, including vascular, neurological, cardiac, and renal impairments. Consequently, individuals with T2DM often have reduced participation in both basic and instrumental activities of daily living, leading to an increase rate of disability- adjusted life years among those affected. Considering this, T2DM imposes a substantial burden not only on healthcare systems but also on society [1,2].

The formation of advanced glycation end products (AGEs), promoted by high glucose levels, accumulates in tissues and leads to endothelial dysfunction, inflammation, oxidative stress, and fibrosis, significantly contributing to DM complications, particularly CV ones. Effective medication management is crucial for addressing DM complications and mitigate their impact. For comprehensive management, medication must be combined with physical activity and diet, all cantered around the patients’ needs, with the ultimate goal of improving their quality of life [2,3,4]. 

Patients with poorly controlled T2DM often present with microvascular complications that affect small blood vessels, such as chronic kidney disease (CKD), diabetic neuropathy, and retinopathy, all of which lead to increased mortality and morbidity rates in this category of patients [5]. CKD is the first cause of kidney failure or kidney transplant among patients with DM, retinopathy is the leading cause of vision-threatening, while diabetic neuropathy can lead to foot ulcers and amputations [6,7,8].

CVD exacerbates macrovascular complications and maintains a bidirectional relationship with T2DM [9]. In addition, several complications can arise from T2DM that make it difficult for most patients to achieve their treatment goals. These complications include conditions like peripheral artery disease, coronary heart disease, cardiomyopathy, arrhythmias, and cerebrovascular disease. They are often caused by risk factors such as high blood pressure (BP), dyslipidemia, and obesity, which not only worsen the condition but also lead to disabilities [10].

Polypharmacy in patients with T2DM is due to the disease itself, along with complications and comorbidities that should be addressed. Moreover, the association between CVD and T2DM highlights the importance of comprehensive care and management strategies targeting both DM and CV risk factors [11]. All these medications add up with the consequences of long-term hyperglycemic status and can lead to non-adherence, keeping the patients from attaining their therapeutic goals, so there is a severe need to integrate new medications into the existing schemes [12]. In addition to polypharmacy, incorporating non-pharmacological strategies such as nutritional therapy, psychological interventions, physical therapies, social interventions, self-blood glucose monitoring in non-insulin-treated T2DM, health coaching, and usual care can improve medication and associated treatment adherence. These interventions can potentially lower CVD risk and serve as adjunctive measures in managing T2DM, enhancing functioning and reducing disability [13]. Knowing that obesity is a significant CV risk factor, the scientific world is investigating incretin role in weight management, starting with glucagon-like peptide-1 receptor agonists (GLP-1 Ra) [14,15,16,17,18,19,20], to dual incretin tirzepatide in SURMOUNT program [21], to the GLP-1 and glucagon receptor agonist cotatutide [22], or even the triple incretin retatrutide [23], and the first results are promising in how clinicians could take action in the obesity pandemic.

Over the years, various classes of medications have been developed to better manage the course of T2DM. Some of these medications were designed to reduce body weight (BW) by suppressing the central sensation of hunger. However, they were later withdrawn from the market due to insufficient data on their long-term CV safety and the lack of evidence of CV benefits [24,25]. The first classes of incretins were dipeptidyl peptidase four inhibitors (DPP-4i) and GLP-1 Ra, and the latter has demonstrated CV benefits through different mechanisms, including BW loss, BP reduction, lipid levels amelioration, and endothelial function improvement [26]. Their benefits were observed in patients with T2DM, with or without obesity, highlighting the newly demonstrated CV benefits along with significant BW reduction (BWR) [27]. Newly described agents, such as the dual glucose-dependent insulinotropic polypeptide (GIP) and GLP-1 Ra tirzepatide, demonstrated significant BWR and better metabolic control compared to other treatment options, including semaglutide, insulin degludec, and insulin glargine [28,29,30]. The future is still under construction with the development of the triple hormone receptor agonist retatrutide (LY3437943), which is a GIP agonist, GLP-1 Ra, and glucagon receptor agonist, and shows promise as an upgrade to the available treatment options, while in a phase 2 trial, it demonstrated remarkable BWR compared to placebo in a dose-dependent manner [23].

**Figure 1:**
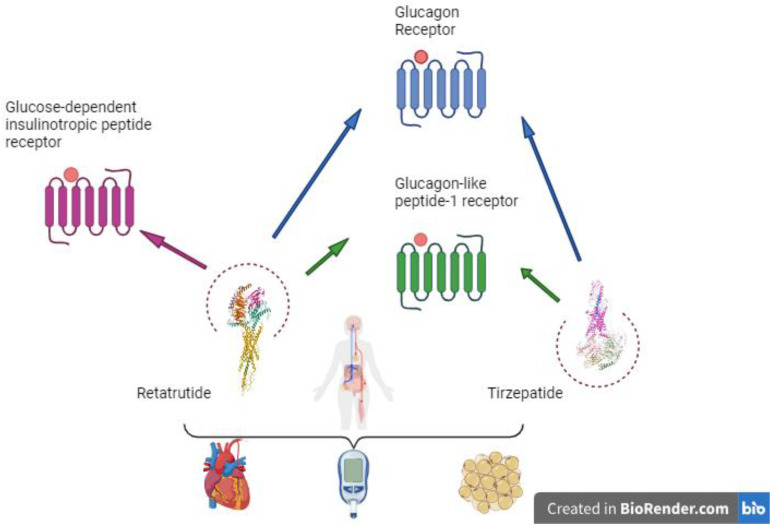
Retatrutide and its potential benefits. Created in BioRender.com.

This systematic review intends to identify the effect of new incretin therapies on CV risk in individuals with DM. For this purpose, we have compared tirzepatide, an incretin that has shown CV benefits in recent studies, with retatrutide, a molecule that is currently being researched in phase 2 studies and awaiting approval, as seen in Figure 1. 

## 2. Results

This early systematic review encompasses seven studies published within the last 3 years. Table 1 summarizes the information extracted from the selected studies, as described below.

The study heterogeneity is emphasized by the comparison between retatrutide and older GLP-1 Ras, such as dulaglutide, the placebo control group and tirzepatide with placebo, and with glargine insulin, following the various duration of follow-up ranging from 10.2 to 72 weeks. The study samples include relatively young patients, with a mean age of 59.39 ± 2.05 years.

Glycated A1c hemoglobin (HbA1c) and BW were assessed in all studies. Other parameters evaluated only in some studies were fasting plasma glucose (FPG), systolic BP (SBP), diastolic BP (DBP), heart rate (HR), and BW, while parameters such as fasting triglycerides (TG), high-density lipoprotein cholesterol (HDL-C), non-high-density lipoprotein cholesterol (non-HDL-C), 10-year predicted atherosclerotic CVD risk score, estimated glomerular filtration rate (eGFR) decline per year, and urine albumin–creatinine ratio (uACR) change from baseline were evaluated in only one study each.

### 2.1. Comparative Dosage Strategies in Retatrutide and Tirzepatide Studies

The studies incorporating retatrutide employed specific dosage strategies, categorizing participants into groups based on their retatrutide dosage. In the study by Doggrell et al. [31,32], participants were allocated diverse doses of retatrutide (1, 2, or 4 mg) that were progressively escalated to a maximum of 8 or 12 mg or a placebo. This diversified dosage strategy aimed to assess the impact of different retatrutide doses on the study participants.

In contrast, Rosenstock et al. [33], divided the patients into various treatment groups, ensuring an even distribution. These groups included placebo, 1.5 mg dulaglutide, and various retatrutide dosages: 0.5 mg, 4 mg escalation, 4 mg, 8 mg slow escalation, 8 mg fast escalation, and 12 mg escalation.

The dosage strategies employed in studies utilizing tirzepatide were structured as follows: in the study led by Hankosky et al. [34], participants underwent random allocation to receive subcutaneous tirzepatide, with dosages ranging between 5, 10, 15 mg, or a placebo. The protocol involved gradually increasing the dosage at 72 weeks, with the medication being administered once weekly. Similarly, Garvey et al. [35], initiated participants with tirzepatide or a placebo, beginning with a weekly dosage of 2.5 mg, that progressively increased by 2.5 mg every four weeks until reaching the targeted dose of either 10 mg or 15 mg at 12 and 20 weeks, respectively. Heerspink et al. [36], randomized participants in a 1:1:1:3 ratio involving tirzepatide dosages of 5 mg, 10 mg, or 15 mg, along with insulin glargine. The study format accounted for differences in dosing schedules between once-per-week tirzepatide and once-per-day insulin glargine. Del Prato et al. [30], utilized random assignment (1:1:1:3) to evaluate tirzepatide at 5 mg, 10 mg, and 15 mg alongside insulin glargine. 

The consistent approach to tirzepatide dosing across these studies facilitates potential comparative analyses and enhances the generalizability of findings.

### 2.2. Side Effects in Incretin Therapy

The most notable side effects in patients treated with incretin therapies were gastrointestinal (GI) ones [17,19,22,28,33,35,37]. STEP trials noted the most frequent adverse events (AE) were nausea, diarrhea, vomiting, and constipation, with the majority being transient and of mild or moderate severity [37]. SCALE trial also reported GI AEs in patients with T2DM treated with injectable liraglutide versus placebo as an add-on to insulin basal therapy (71% vs. 49%), the most frequent being nausea, vomiting, constipation, diarrhea, or abdominal discomfort [19]. SURPASS-2, a phase 3 trial, demonstrated the superiority of tirzepatide, compared to semaglutide in patients with T2DM, with an elevated rate of mild to moderate GI AE in the tirzepatide vs. semaglutide group (nausea, 17 to 22% and 18%; diarrhea, 13 to 16% and 12%; and vomiting, 6 to 10% and 8%, respectively), with a greater AE risk in the 15 mg group compared to the 10 mg, 5 mg, or semaglutide group [28]. Cotadutide, a dual GLP-1 RA and glucagon receptor agonist was also associated with GI AEs when compared to liraglutide 1.8 mg or placebo, and the risk was proportional to the dose escalation [22]. Also, the triple incretin therapy, retatrutide, has been reported to have mild to moderate GI side effects, and the proportion of nausea, vomiting and constipation was elevated in the escalated dosage group (13% vs. 50% in 0.5 mg group compared to the 8 mg one) [33].

## 3. Discussion

GLP-1 Ra agents demonstrated CV benefits in several trials, including semaglutide, both in injectable [38] and oral administration [39], liraglutide [40], and dulaglutide [41]. These trials pioneered the CV protection point of view, a required attribute for every new antidiabetic medication.

Currently, injectable semaglutide is the only GLP-1Ra that has an additional approved indication for the reduction in major adverse CV events in adults, with established CVD and either overweight or obesity, approved by United States Food and Drug Administration. The approval decision was supported by findings from the placebo-controlled SELECT trial, which showed that the injectable semaglutide given on top of standard therapy significantly reduced the risk of CV death, myocardial infarction, or stroke (6.5% vs. 8.0%; hazard ratio 0.80; 95% confidence interval 0.72–0.90) in patients with established CVD, no prior history of DM, and a body mass index of at least 27 kg/m^2^. The exact mechanism of the CV risk reduction observed in the trial is unclear [42].

Because incretins significantly reduced CV events (CVEs), researchers have searched for the physiopathology behind these benefits, considering their metabolic implications [43]. Tuttolomondo et al. demonstrated that dulaglutide significantly decreased DBP, BW, total cholesterol and low-density lipoprotein cholesterol (LDL-C), FPG, HbA1c, microalbuminuria, and pulse wave velocity, while significantly increasing the reactive hyperemia index after nine-month follow-up in patients with T2DM compared to traditional antidiabetic treatment alone [44]. All these parameters represent valuable markers of vascular health, with effects on endothelial and arterial stiffness indexes. A Study of Tirzepatide (LY3298176) Compared with Dulaglutide on Major Cardiovascular Events in Participants with Type 2 Diabetes (SURPASS-CVOT) is an ongoing trial trying to evaluate the noninferiority or even superiority of the dual incretin, tirzepatide, in comparison to the GLP-1 Ra dulaglutide in terms of major adverse CVEs in patients with T2DM [45]. These research trends emphasize the importance of better understanding CV benefits in novel antidiabetic therapies.

Another phase 2b placebo-controlled trial, which investigated an oral GLP-1 Ra, danuglipron, demonstrated improved HbA1c, basal glycemia, and BWR in patients with T2DM in the active group [46]. Further studies are needed to determine whether this new oral GLP-1 Ra also has CV protection or not in future trials with well-established CV outcomes.

The studies evaluating retatrutide and tirzepatide are either in phase 1 or phase 2, emphasizing their developmental stages where safety and efficacy are assessed. However, tirzepatide is in a phase 3 trial, being compared to placebo or glargine insulin, reflecting its comprehensive evaluation, pivotal for regulatory considerations, and potential approval. This distinction underscores the evolving nature and maturity of the investigated therapies.

The variety of study durations observed ranges from 10.2 to 72 weeks, enabling researchers and clinicians to assess the subtleties of these interventions, potentially revealing trends, sustained benefits, and emerging long-term outcomes. This consideration is especially relevant in chronic conditions such as DM, where long-term management and sustained efficacy are crucial. 

Moreover, the mean age of 59.39 ± 2.05 years reflects young participants, an age demographic group that is facing increased challenges in DM management, making the outcomes particularly relevant for understanding the effectiveness of retatrutide and tirzepatide in addressing the specific needs and complexities associated with an older age group.

The consistent use of tirzepatide doses across Hankosky et al. [34], Heerspink et al. [36] and Del Prato et al. [30] study reflects a standardized approach in their respective evaluations, facilitating potential comparative analyses and enhances the generalizability of the findings.

The efficacy results on HbA1c for retatrutide [31,32,33], compared to other antidiabetic classes or placebo, and for tirzepatide compared to placebo [28] or insulin [30], were also observed for the most recent once-weekly GLP-1 and glucagon receptor dual agonist, mazdutide. These results are also present for BWR. So, the efficacy is not limited to race, as mazdutide is being evaluated on Chinese patients, with T2DM leading the way to future research on CV protection in patients treated with dual incretin therapies [47]. Other adjuvants of the antidiabetic treatment with benefits in CV protection are supplements that are reported to lower FPG levels and protein glycation (AGEs and HbA1c), ameliorates LDL-C level, and improve HDL-C functionality [48,49]. If we talk about the HbA1c value, in our study, for retatrutide, it ranges between 1.9 and 2.16%, which is inferior to the value for tirzepatide of 2.1–2.58% and superior to GLP-1 Ra −0.78–1.9%. For BW, retatrutide with 9–17.87 kg is like tirzepatide with 11.7–14.8 kg, but higher than GLP-1 Ra with 0.64–5.8 kg [50]. Similar data are reported for the FPG, which, in our study, is decreased by retatrutide and also for tirzepatide, with a greater effect for the first class.

Moreover, for BP, in our study, the reduction in SBP ranges between 8.3 and 12.1 mmHg for retatrutide, which is superior to the reduction of 4.8–6.3 mmHg for tirzepatide. Similarly, the effect on DBP of 4.6–8.1 mmHg was superior to the reduction of 0.7–2.5 mmHg for tirzepatide; retatrutide demonstrated a reduction in HR of 6.7 beat per minutes, which is higher than 4.1 beat per minutes for tirzepatide.

Regarding the lipidic profile, there are reported data about a decrement of LDL-C, VLDL-C, TG, and HDL-C only for retatrutide. Data that are reported only for tirzepatide are about its efficacy in reducing the 10-year predicted ASCVD risk score; the level of fasting TG, HDL-C, and non-HDL-C; eGFR decline per year; and uACR change from baseline.

Our study limitations are linked to the scarcity of data on long-term CV protection for these new dual and triple incretins. These agents need longer follow-up trials to establish their benefit in reducing the risk for major adverse CVEs. Also, an indirect limitation is that the included studies design does not provide a distribution of the drug’s effects regarding patient’s parameters, such as BMI. These new data could be a promising research area for future investigations. Also, both tirzepatide and retatrutide can be administered through injections, which could represent a barrier to the patient’s adherence in the first place despite their promising CV benefits.

Furthermore, the Semaglutide Effects on Cardiovascular Outcomes in People with Overweight or Obesity (SELECT) trial, a randomized controlled trial that evaluates the CV benefit of a once-weekly injection of semaglutide in patients with overweight or obesity but without T2DM, will lead to a more comprehensive interpretation of incretins’ CV effects, unattached to the metabolic improvement in patients with T2DM, which can represent a major confounding factor [41].

On the other hand, the trial extension with once-weekly Semaglutide in overweight or obese adults (STEP-1) found that participants regained two-thirds of their BWR, with similar results in other cardiometabolic parameters, such as lipid profile, C-reactive protein levels, or BP values, after one year of withdrawal from semaglutide [51,52]. These findings emphasize the complexity and chronic character of obesity and its involvement in CV risk modulation [53,54].

These CV benefits, the reason why the GLP-1 Ra class is recommended, have been researched in several real-life studies. Liraglutide and semaglutide (the injectable administration form) demonstrated significant effects in reducing carotid intima-media thickness and lipid profile amelioration, in addition to their effects on glucose metabolism [29,34]. These results emphasize the need for this class in the early stages of CVD to achieve the best results in preventing CVEs.

Despite the well-known CV protective effects of this therapy class, some patients still do not achieve their therapeutic targets, partly because of therapeutic inertia, and partly because of their unavailability in different areas, including developing countries [3,55,56]. These latest data should raise awareness of the importance of CV risk stratification and proper therapeutic approaches, in accordance with guidelines. New molecules included in the incretins class could represent a chance for patients with T2DM to achieve their metabolic goals and reduce their CV risk.

## 4. Materials and Methods

The present systematic review followed the Preferred Reporting Items for Systematic Reviews and Meta-Analysis (PRISMA) checklist and guidelines. The review protocol has been registered with the identifier CRD42024507397.

### 4.1. Research Question and Search Strategy

An electronic search for relevant publications was performed using PubMed and Web of Science library databases and was conducted from January 2013 to February 2024. The following search strategy was used: Retatrutide or Tirzepatide and cardiovascular disease and (diabetes mellitus or type 2 diabetes mellitus OR T2DM). After this search, 93 articles were found (63 from PubMed and 30 from Web of Science). After applying filters for language (English), publication type (original articles), and date range (2013 to the date of the search), 15 articles remained. These articles underwent initial title screening, followed by an abstract review by two independent reviewers, and 13 articles remained for a full assessment.

Because the titles and abstracts lacked data about the retatrutide molecule, we performed another search of PubMed and Web of Science library databases with “retatrutide” and obtained 48 articles (26 on PubMed and 22 on Web of Science, respectively). After applying filters for language (English) and publication type (original articles), 9 articles remained. After removing the duplicates, 7 articles remained for full assessment.

**Figure 2:**
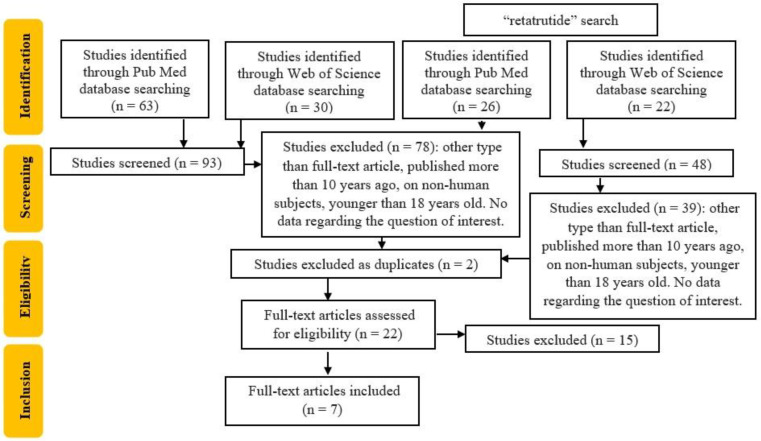
Flowchart of the study selection.

As seen in Figure 2, 7 studies were included from the 20 articles that were fully assessed.

The research question was framed using the Population, Intervention, Comparison, and Outcome (PICO) method. The population was represented by patients with T2DM and CV risk, and intervention was represented by the new incretin therapy (tirzepatide and retatrutide). The comparison was against patients who received treatment other than the agent of interest. The outcome was defined by BW, HbA1c, FPG level, lipid profile, BP, and HR or other CV risk benefits in patients with T2DM and new incretin therapy, and the effect was measured by percentage, confidence intervals, odds ratios, and relative risk.

### 4.2. Inclusion Criteria

The studies had to meet specific publication criteria to be included in this review. These criteria are as follows: (i) the studies should be original full-text articles that are randomized control or clinical trials; (ii) the articles should be published within the last ten years; (iii) the articles should be published in English; and (iv) the studies should be conducted on adult human populations.

### 4.3. Exclusion Criteria

Studies were excluded from the analysis if they were deruled on (i) cell cultures or (ii) mammals.

### 4.4. Selection of Studies

Studies that met the following eligibility criteria were selected: (1) included patients with T2DM and CV risk; (2) patients received either tirzepatide or retatrutide; (3) provided sufficient information such as the corresponding 95% CIs or at least *p*-value. Studies were excluded if they (1) were a letter to the editor, expert opinions, case reports, meeting abstracts, or reviews; (2) were redundant publications; or (3) needed more precise or complete data.

### 4.5. Data Extraction

Two authors (T.S. and C.-G.P.) used a self-made data extraction table to individually evaluate and extract the following data for each included article: the first author and year of publication, study period, sample size, the average age of participants, treatment details, outcomes, confounding factors adjusted, reported outcomes, and risk estimates with their corresponding 95% CIs. Whenever there were differences in opinion, we resolved them by having a discussion or by consulting with a third author (I.-C.B.).

### 4.6. Risk of Bias Assessment

Two reviewers (T.S. and C.-G.P.) independently assessed the quality of the studies according to Newcastle–Ottawa Scale criteria and provided their classification in Table 2.

### 4.7. Strategy for Data Synthesis 

A narrative synthesis of the findings in the studies, centered around each class of incretin therapy and benefit on CV risk, where a minimum of two studies were identified, was performed.

## 5. Conclusions

DM is a disease that can have serious CV, renal, nervous, and ophthalmic complications if it is not diagnosed and treated adequately. For this reason, it is essential to follow a therapy that aims to maintain blood glucose levels within normal values. In recent years, new innovative drugs have been introduced on the market for the treatment of T2DM, which have been shown to have beneficial effects not only on glycemic control, but also on the prevention of CV complications.

A new molecule, called tirzepatide, has recently been identified, capable of acting simultaneously on the GLP-1 and GIP receptors, thus making its hypoglycemic activity even more effective compared to drugs already in use. Furthermore, a characteristic of this class of drugs is that they also achieve a significant BWR, which is why they have also been studied as a treatment for obesity in patients both with and without T2DM.

In the study phase, we have retatrutide, characterized by a more complex mechanism of action than its potential competitors as it acts on three receptor targets: GIP, GLP-1, and glucagon. Its action as a triple receptor agonist has proven effective in inducing BWR and metabolic effects with results comparable to those of bariatric surgery.

These drugs represent a new frontier in the treatment of T2DM, as they allow us to modify the course of the disease and aim for its remission. In fact, some studies have shown that these drugs are able to preserve or improve the function of pancreatic beta cells, reduce chronic inflammation, and promote the regeneration of tissues damaged by DM. In this way, functional healing and a reduction in long-term complications could be achieved. The arrival of new innovative therapies offers new opportunities and hopes to patients with T2DM, who can thus pursue the goal of better quality and greater life expectancy. Much research still needs to be conducted to apply these therapies in a personalized manner to the various metabolic alteration phenotypes.

## Figures and Tables

**Table 1:** Included studied characteristics and parameters of interest.

First Author, Year of Publication	Trial Phase	Duration of the Study	Sample Size	Mean Age	Treatment Arm Comparator	Clinical Outcome	Statistical Power
Doggrell et al. [31], 2023	2	24 weeks	281	56.2	Retatrutide 12 mg	HbA1c = −2.02%	
					Dulaglutide 1.5 mg	HbA1c = −1.41%	
					Placebo group	HbA1c = −0.01%	
					Retatrutide 12 mg	BWR = NR, but differences were registered	
					Dulaglutide 1.5 mg	No effect of BWR	
					Placebo group	BWR NR	
					Retatrutide 12 mg	SBP (−8.3, −12.1)/DBP (−4.6, −8.1) mmHg	
					Dulaglutide 1.5 mg	SBP/DBP reduction NR	
					Placebo group	SBP/DBP reduction NR	
					Retatrutide 12 mg	HR = −6.7 beat/min	
					Dulaglutide 1.5 mg	HR reduction NR	
					Placebo group	HR reduction NR	
					Retatrutide 12 mg	↓ LDL-C ↓ VLDL-C ↓ TG ↑ HDL-C	
					Dulaglutide 1.5 mg	LDL-C, VLDL-C, TG, HDL-C differences NR	
					Placebo group	LDL-C, VLDL-C, TG, HDL-C differences NR	
Doggrell et al. [32], 2023	1b	78 days	34	NR	Retatrutide 12 mg	HbA1c = −1.9%	
					Dulaglutide 1.5 mg	HbA1c = −1%	
					Retatrutide 12 mg	BWR = −9kg	
					Dulaglutide 1.5 mg	BWR NR	
					Retatrutide 12 mg	↓ LDL-C ↓ VLDL-C ↓ TG ↓ HDL-C	
					Dulaglutide 1.5 mg	LDL-C, VLDL-C, TG, HDL-C differences NR	
					Retatrutide 12 mg	↓ SBP ↓ DBP ↑ HR	
					Dulaglutide 1.5 mg	SBP, DBP, HR differences NR	
Rosenstock et al. [33], 2023	2	36 weeks	281	56.2	Retatrutide 12 mg	HbA1c = −2.16%	*p* < 0.0001
					Dulaglutide 1.5 mg	HbA1c = −1.36%	*p* < 0.0001
					Placebo group	HbA1c = −0.3%	*p* = 0.2091
					Retatrutide 12 mg	FPG = −67.87 mg/dL	*p* < 0.0001
					Dulaglutide 1.5 mg	FPG = −27.53 mg/dL	*p* = 0.0024
					Placebo group	FPG = −17.26 mg/dL	*p* = 0.1126
					Retatrutide 12 mg	BWR = −17.18 kg	*p* < 0.0001
					Dulaglutide 1.5 mg	BWR = −1.97 kg	*p* = 0.0242
					Placebo group	BWR = −3.28 kg	*p* = 0.0004
Hankosky et al. [34], 2023		72 weeks	2539	44.5 ± 12.3	Tirzepatide 15 mg	10-year predicted ASCVD risk score variability = −22.4%	OR 2.4, 95% CI (1.7, 3.5), *p* < 0.001
					Placebo	10-year predicted ASCVD risk score variability = 12.7%	
Garvey et al. [35], 2023	3	72 weeks	938	54.2	Tirzepatide 15 mg	BWR = −14.8 kg	−11.6%, 95% CI (−13, −10.1, *p* < 0.0001)
					Placebo	BWR = −3.2 kg	
					Tirzepatide 15 mg	HbA1c = −2.1% ± 0.07	*p* < 0.0001
					Placebo	HbA1c = –0.5% ± 0.07	
					Tirzepatide 15 mg	SBP = −6.3 mmHg	*p* < 0.0001
					Placebo	SBP = −1.2 mmHg	
					Tirzepatide 15 mg	DBP = −2.5 mmHg	*p* = 0.0012
					Placebo	DBP = −0.3 mmHg	
					Tirzepatide 15 mg	Fasting TG = −27.2%	*p* < 0.0001
					Placebo	Fasting TG = −3.3%	
					Tirzepatide 15 mg	HDL-C = −9%	*p* < 0.0001
					Placebo	HDL-C = −0.2%	
					Tirzepatide 15 mg	Non-HDL-C = −5.9%	*p* < 0.0001
					Placebo	Non-HDL-C = 3.7%	
Heerspink et al. [36], 2022	3	52 weeks	1995	63.6	Tirzepatide 15 mg	eGFR decline per year = −1.4 mL/min/1.73 m^2^ ± 0.2	2.2 mL/min/1.73 m^2^, 95% CI (1.6, 2.8, *p* = 0.55)
					Glargin insulin	eGFR decline per year = −3.6 mL/min/1.73 m^2^ ± 0.2	
					Tirzepatide 15 mg	uACR change from baseline = −6.8%, 95% CI (−14.1, 1.1)	−31.9%, 95% CI (−37.7, −25.7, *p* = 0.99)
					Glargin insulin	uACR change from baseline = 36.9%, 95% CI (26, 48.7)	
Del Prato et al. [30], 2021	3	52 weeks	1995	63.6	Tirzepatide 15 mg	HbA1c = −2.58 ± 0.05%	
					Glargin insulin	HbA1c = −1.44 ± 0.03%	
					Tirzepatide 15 mg	BWR = −11.7 ± 0.33	
					Glargin insulin	BWR = −1.9 ± 0.19	
					Tirzepatide 15 mg	FPG = −3.29 ± 0.115	
					Glargin insulin	FPG = −2.84 ± 0.066	
					Tirzepatide 15 mg	SBP = −4.8 ± 0.74	
					Glargin insulin	SBP = −1.3 ± 0.44	
					Tirzepatide 15 mg	DBP = −1 ± 0.44	
					Glargin insulin	DBP = −0.7 ± 0.26	
					Tirzepatide 15 mg	HR = −4.1 ± 0.48	
					Glargin insulin	HR = −1.2 ± 0.29	

HbA1c—glycated hemoglobin A1C, BWR—body weight reduction, SBP—systolic blood pressure, DBP—diastolic blood pressure, HR—heart rate, LDL-C—low-density lipoprotein cholesterol, VLDL-C—very low-density lipoprotein cholesterol, HDL-C—high-density lipoprotein cholesterol, TG—triglycerides, non-HDL-C—non-high-density lipoprotein cholesterol, FPG—fasting plasma glucose, ↓—a decrease was reported; ↑—an increase was reported; ASCVD—atherosclerotic cardiovascular disease, eGFR—estimated glomerular filtration rate, uACR—urine albumin-creatinine ratio.

**Table 2:** Newcastle–Ottawa Scale analysis of the included articles.

Author (Reference)	Selection				Comparability	Outcome			Total Score	Quality
	Representativeness of the Exposed Cohort	Selection of the Non-Exposed Cohort	Ascertainment of Exposure	Demonstration That Outcome of Interest Was Not Present at the Start of the Study	Comparability of Cohorts Based on the Design or Analysis	Assessment of Outcome	Was Follow-Up Long Enough for Outcomes to Occur	Adequacy of Follow-Up of Cohorts		
Doggrell et al. [31], 2023	*	-	*	*	-	*	*	*	6	good
Doggrell et al. [32], 2023	*	-	*	*	-	*	*	*	6	good
Rosenstock et al. [33], 2023	-	*	*	*	*	*	*	*	7	good
Hankosky et al. [34], 2023	-	-*	*	*	*	*	*	*	7	good
Garvey et al. [35], 2023	-	*	*	*	*	*	*	*	7	good
Heerspink et al. [36], 2022	-	*	*	*	*	*	*	*	7	good
Del Prato et al. [30], 2021	-	*	*	*	*	*	*	*	7	good

“*” indicates that the article meets the criteria mentioned above; “-” indicates that the article does not meet the abovementioned criteria.
